# Supplementary material for: Effect of dark sweet cherry powder consumption on the gut microbiota, short-chain fatty acids, and biomarkers of gut health in obese db/db mice
Source: PeerJ. 2018 Jan 3;6:e4195. doi: 10.7717/peerj.4195 (PMC5756454; doi:10.7717/peerj.4195)
Supplement: Supplemental Information 1 [file peerj-06-4195-s001.docx]

**Effect of dark sweet cherry powder consumption on the gut microbiota, short-chain fatty acids, and biomarkers of gut health in obese db/db mice (SUPPLEMENTARY INFORMATION)**

Jose F. Garcia-Mazcorro^1,2^, Nara Nunes Lage^3,4^ Susanne Mertens-Talcott^4^, Stephen Talcott^4^, Boon Chew^4^, Scot E. Dowd^5^, Jorge R. Kawas^2,6^, and Giuliana D. Noratto^4^

^1^Faculty of Veterinary Medicine, Universidad Autonoma de Nuevo Leon (UANL), General Escobedo, Nuevo Leon, Mexico

^2^MNA de Mexico, San Nicolas de los Garza, Nuevo Leon, Mexico

^3^Research Center in Biological Sciences, Federal University of Ouro Preto, Minas Gerais, Brazil

^4^Department of Nutrition and Food Science, Texas A&M University, College Station, Texas, USA

^5^Molecular Research LP, Shallowater, Texas, USA

^6^Faculty of Agronomy, UANL, General Escobedo, Nuevo Leon, Mexico

Corresponding: Giuliana Noratto, [gnoratto@tamu.edu](mailto:gnoratto@tamu.edu)

**Supplementary Figures**


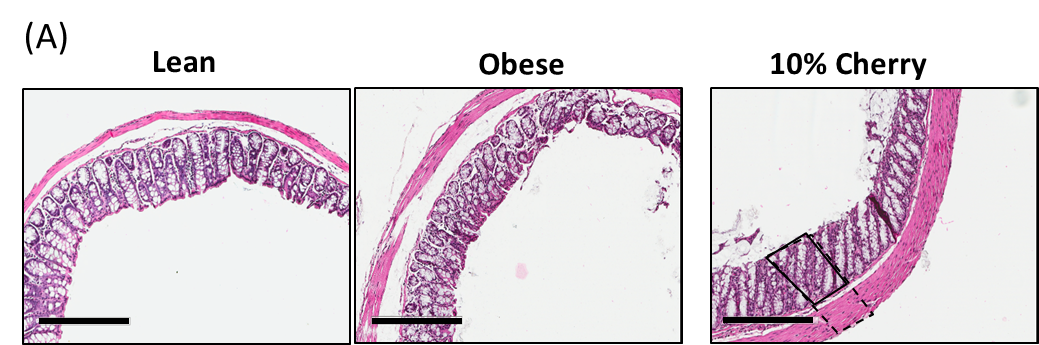

**Supplementary Figure S1.** Cherry intake improved colon barrier measured through area of outer colon wall (continue line box) relative to total area colon wall (continue and dashed line boxes). (A) Representative photomicrographs taken from colon sections stained with H&E. Bar = 300 µm, 20X. Mice fed control diet (lean and obese) or cherry supplemented diet (10%). Photomicrographs were taken with Aperio CS2 digital pathology scanner (Leica Biosystems Inc. Buffalo Grove, IL). (B) Quantitative results of outer colon layer relative to total colon wall area. Photomicrographs were blinded analyzed with ImageJ software (http://rsb.info.nih.gov/ij/). Areas were measured along the colon tissue (10 or more measurements each picture) from different animals (n ≥ 5). Box plots represent median (line inside the box) and whiskers (min to max). Data was analyzed with Kruskal-Wallis test followed by Dunn’s multiple comparison test (p = 0.08) using GraphPad Prism 5.01 Software Inc.

**
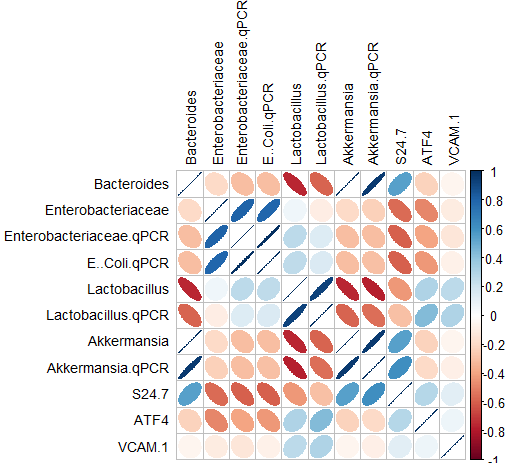
**

**Supplementary Figure S2.** Spearman’s correlation matrix of fecal bacteria versus end point biomarkers of intestinal health (mRNA levels of ATF4 and VCAM-1 in colonic mucosal cells). The direction of ellipses represents positive or negative correlations and the width of ellipses represents the strength of correlation (narrow ellipse = stronger correlation).

**Supplementary Tables**

**Supplementary Table S1.** Nutritional contents of cherry powder.

| **Cherry powder** | | **Content (g/100 g powder)** |
| --- | --- | --- |
| Protein | | 4.2% |
| Ash | | 4.8% |
| Moisture | | 3.6% |
| Total dietary fiber | | 5.1% |
| Maltodextrin | | 20% |
| Silicon dioxide | | 2% |
| Sugars | Fructose | 20.4% |
|  | Glucose | 35% |
|  | Lactose | < 0.1% |
|  | Maltose | 1.3% |
|  | Sucrose | < 0.1% |
| Total extractable phenolics (mg GAE/100 g) | | 629 ± 39 |
| Total bound non-extractable phenolics (mg GAE/100 g) | | 130 ± 3.9 |

Dark sweet cherries (Bing variety) were processed and freeze dried by Powder Pure (The Dalles, OR) to obtain cherry powder used for mice diet.

**Supplementary Table S2.** Targets, primers and references for all bacterial groups use in qPCR.

| Target | Primer sequence (5’-3’) | Reference |
| --- | --- | --- |
| Firmicutes | TGAAACTYAAAGGAATTGACG  ACCATGCACCACCTGTC | Bacchetti De Gregoris et al. (2011) |
| *Lactobacillus* spp. | AGCAGTAGGGAATCTTCCA  CACCGCTACACATGGAG | Walter et al. (2001), Heilig et al. (2002) |
| *Lactobacillus plantarum* | CTCTGGTATTGATTGGTGCTTGCAT  GTTCGCCACTCACTCAAATGTAAA | Matsuda et al. (2009) |
| *Lactobacillus acidophilus* | GCAGATCGCATGATCAGCTTATA  TCAGTCTCTCAACTCGGCTATG | Firmesse et al. (2008) |
| Ruminococcaceae | ACTGAGAGGTTGAACGGCCA  CCTTTACACCCAGTAAWTCCGGA | Garcia-Mazcorro et al. (2012) |
| *Faecalibacterium* | GAAGGCGGCCTACTGGGCAC  GTGCAGGCGAGTTGCAGCCT | Garcia-Mazcorro et al. (2012) |
| *Clostridium butyricum* | GTGCCGCCGCTAACGCATTAAGTAT  ACCATGCACCACCTGTCTTCCTGCC | Bartosch et al. (2004) |
| *Clostridium* cluster IV (*C. leptum* group) | GCACAAGCAGTGGAGT  CTTCCTCCGTTTTGTCAA | Matsuki et al. (2004) |
| *Eubacterium halii* | GCGTAGGTGGCAGTGCAA  GCACCGRAGCCTATACGG | Ramirez-Farias et al. (2009) |
| *Enterococcus* | CCCTTATTGTTAGTTGCCATCATT  ACTCGTTGTACTTCCCATTGT | Rinttilä et al. (2004) |
| *Turicibacter* | CAGACGGGGACAACGATTGGA  TACGCATCGTCGCCTTGGTA | Suchodolski et al. (2012) |
| CFB (Cytophaga-Flavobacterium-Bacteroides phylum) | CCGGAWTYATTGGGTTTAAAGGG  GGTAAGGTTCCTCGCGTA | Mühling et al. (2008) |
| Bacteroidetes | GGARCATGTGGTTTAATTCGATGAT  AGCTGACGACAACCATGCAG | Guo et al. (2008) |
| *Bacteroides/Prevotella* | GAGAGGAAGGTCCCCCAC  CGCTACTTGGCTGGTTCAG | Layton et al. (2006) |
| *Bacteroides* spp. | CGATGGATAGGGGTTCTGAGAGGA  GCTGGCACGGAGTTAGCCGA | Bergström et al. (2012) |
| *Bacteroides fragilis* | CTGAACCAGCCAAGTAGCG  CCGCAAACTTTCACAACTGACTTA | Liu et al. (2003) |
| *Bacteroides vulgatus* | GCATCATGAGTCCGCATGTTC  TCCATACCCGACTTTATTCCTT | Wang et al. (1996) |
| *Bacteroides thetaiotaomicron* | GGCAGCATTTCAGTTTGCTTG  GGTACATACAAAATTCCACACGT | Wang et al. (1996) |
| *Bacteroides eggerthi* | GTCATATTAACGGTGGCG  GGGTTBCCCCATTCGG | Liu et al. (2003) |
| *Parabacteroides distasonnis* | TGATCCCTTGTGCTGCT  ATCCCCCTCATTCGGA | Liu et al. (2003) |
| Betaproteobacteria | AACGCGAAAAACCTTACCTACC  TGCCCTTTCGTAGCAACTAGTG | Yang et al. (2015) |
| *Bifidobacterium* | GCGTGCTTAACACATGCAAGTC  CACCCGTTTCCAGGAGCTATT | Penders et al. (2005) |
| *Bifidobacterium adolescentes* | CTCCAGTTGGATGCATGTC  CGAAGGCTTGCTCCCAGT | Matsuki et al. (1998) |
| *Bifidobacterium breve* | AATGCCGGATGCTCCATCACAC  GCCTTGCTCCCTAACAAAAGAGG | Rinne et al. (2005) |
| Enterobacteriaceae | CATTGACGTTACCCGCAGAAGAAGC  CTCTACGAGACTCAAGCTTGC | Bartosch et al. (2004) |
| *E. coli* | CATGCCGCGTGTATGAAGAA  CGGGTAACGTCAATGAGCAAA | Huijsdens et al. (2002) |
| *Desulfovibrio* | CCGTAGATATCTGGAGGAACATCAG  CCGTAGATATCTGGAGGAACATCAG | Fite et al. (2004) |
| *Akkermansia muciniphila* | CAGCACGTGAAGGTGGGGAC  CCTTGCGGTTGGCTTCAGAT | Collado et al. (2007) |
| Deferribacteres | CTATTTCCAGTTGCTAACGG  GAGHTGCTTCCCTCTGATTATG | Yang et al. (2015) |
| Tenericutes | ATGTGTAGCGGTAAAATGCGTAA  CMTACTTGCGTACGTACTACT | Yang et al. (2015) |

**References for Supplementary Table S2.**

**Bacchetti De Gregoris T, Aldred N, Clare AS, Burgess JG. 2011.** Improvement of phylum- and class-specific primers for real-time PCR quantification of bacterial taxa. *Journal of Microbiological Methods* **86:**351-356.

**Bartosch S, Fite A, Macfarlane GT, McMurdo ME. 2004.** Characterization of bacterial communities in feces from healthy elderly volunteers and hospitalized elderly patients by using real-time PCR and effects of antibiotic treatment on the fecal microbiota. *Applied Environmental Microbiology* **70:**3575-3581.

**Bergström A, Licht TR, Wilcks A, Andersen JB, Schmidt LR, Grønlund HA, Vigsnæs LK, Michaelsen KF, Bahl MI. 2012.** Introducing Gut Low-Density Array (GULDA) – a validated approach for qPCR-based intestinal microbial community analysis. *FEMS Microbioloy Letters* **337(1):**38-47.

**Collado MC, Derrien M, Isolauri E, de Vos WM, Salminen S. 2007.** Intestinal integrity and *Akkermansia muciniphila*, a mucin-degrading member of the intestinal microbiota present in infants, adults, and the elderly. *Applied Environmental Microbiology* **73:**7767-7770.

**Firmesse O, Mogenet A, Bresson JL, Corthier G, Furet JP. 2008.** *Lactobacillus rhamnosus* R11 consumed in a food supplement survived human digestive transit without modifying microbiota equilibrium as assessed by real-time polymerase chain reaction. *Journal of Molecular Microbiology and Biotechnology* **14:**90-99.

**Fite A, Macfarlane GT, Cummings JH, Hopkins MJ, Kong SC, Furrie E, Macfarlane S. 2004.** Identification and quantitation of mucosal and faecal desulfovibrios using real time polymerase chain reaction. *Gut* **53:**523-529.

**Garcia-Mazcorro JF, Suchodolski JS, Jones KR, Clark-Price SC, Dowd SE, Minamoto Y, Markel M, Steiner JM, Dossin O. 2012.** Effect of the proton pump inhibitor omeprazole on the gastrointestinal microbiota of healthy dogs. *FEMS Microbiology Ecology* **80:**624-636.

**Guo X, Xia X, Tang R, Zhou J, Zhao H, Wang K. 2008.** Development of a real-time PCR method for Firmicutes and Bacteroidetes in faeces and its application to quantify intestinal population of obese and lean pigs. *Letters in Applied Microbiology* **47:**367-373.

**Heilig HG, Zoetendal EG, Vaughan EE, Marteau P, Akkermans AD, de Vos WM. 2002.** Molecular diversity of Lactobacillus spp. and other lactic acid bacteria in the human intestine as determined by specific amplification of 16S ribosomal DNA. *Applied Environmental Microbiology* **68:**114–123.

**Huijsdens XW, Linskens RK, Mak M, Meuwissen SG, Vandenbroucke-Grauls CM & Savelkoul PH. 2002.** Quantification of bacteria adherent to gastrointestinal mucosa by real-time PCR. Journal of Clinical Microbiology **40:**4423-4427.

**Layton A, McKay L, Williams D, Garrett V, Gentry R, Sayler G. 2006.** Development of Bacteroides 16S rRNA gene TaqMan-based real-time PCR assays for estimation of total, human, and bovine fecal pollution in water. *Applied Environmental Microbiology* **72:**4214-4224.

**Liu C, Song Y, McTeague M, Vu AW, Wexler H, Finegold SM. 2003.** Rapid identification of the species of the *Bacteroides fragilis* group by multiplex PCR assays using group- and species-specific primers. *FEMS Microbiology Letters* **222:**9-16.

**Matsuda K, Tsuji H, Asahara T, Matsumoto K, Takada T, Nomoto K. 2009.** Establishment of an analytical system for the human fecal microbiota, based on reverse transcription-quantitative PCR targeting of multicopy rRNA molecules. *Applied Environmental Microbiology* **75:**1961–1969.

**Matsuki T, Watanabe K, Fujimoto J, Takada T, Tanaka R. 2004.** Use of 16S rRNA gene-targeted group-specific primers for real-time PCR analysis of predominant bacteria in human feces. *Applied Environmental Microbiology* **70:**7220-7228.

**Matsuki T, Watanabe K, Tanaka R, Oyaizu H. 1998.** Rapid identification of human intestinal bifidobacteria by 16S rRNA-targeted species- and group-specific primers. *FEMS Microbiology Letters* 167: 113-121.

**Mühling M, Woolven-Allen J, Colin Murrell J, Joint I. 2008.** Improved group-specific PCR primers for denaturing gradient gel electrophoresis analysis of the genetic diversity of complex microbial communities. *ISME Journal* **2:**379-392.

**Penders J, Vink C, Driessen C, London N, Thijs C, Stobberingh EE. 2005.** Quantification of *Bifidobacterium* spp., *Escherichia coli* and *Clostridium difficile* in faecal samples of breast-fed and formula-fed infants by real-time PCR. *FEMS Microbiology Letters* **243:**141–147.

**Ramirez-Farias C, Slezak K, Fuller Z, Duncan A, Holtrop G, Louis P. 2009.** Effect of inulin on the human gut microbiota: stimulation of *Bifidobacterium adolescentis* and *Faecalibacterium prausnitzii*. *British Journal of Nutrition* **101:**541-550.

**Rinne MM, Gueimonde M, Kalliomaki M, Hoppu U, Salminen SJ, Isolauri E. 2005.** Similar bifidogenic effects of prebiotic-supplemented partially hydrolyzed infant formula and breastfeeding on infant gut microbiota. *FEMS Immunology Medical Microbiology* **43:**59–65.

**Rinttilä T, Kassinen A, Malinen E, Krogius L, Palva A. 2004.** Development of an extensive set of 16S rDNA-targeted primers for quantification of pathogenic and indigenous bacteria in faecal samples by real-time PCR. *Journal of Applied Microbiology* **97:**1166–1177.

**Suchodolski JS, Markel ME, Garcia-Mazcorro JF, Unterer S, Heilmann RM, Dowd SE, Kachroo P, Ivanov I, Minamoto Y, Dillman EM, Steiner JM, Cook AK, Toresson L. 2012.** The fecal microbiome in dogs with acute diarrhea and idiopathic inflammatory bowel disease. *PLoS ONE* **7(12):**e51907.

**Walter J, Hertel C, Tannock GW, Lis CM, Munro K, Hammes WP. 2001.** Detection of Lactobacillus, Pediococcus, Leuconostoc, and Weissella species in human feces by using group-specific PCR primers and denaturing gradient gel electrophoresis. *Applied Environmental Microbiology* **67:**2578-2585.

**Wang RF, Cao WW, Cerniglia CE. 1996.** PCR detection and quantitation of predominant anaerobic bacteria in human and animal fecal samples. *Applied Environmental Microbiology* **62:**1242-1247.

**Yang YW, Chen MK, Yang BY, Huang XJ, Zhang XR, He LQ, Zhang J, Hua ZC. 2015.** Use of 16S rRNA gene-targeted group-specific primers for real-time PCR analysis of predominant bacteria in mouse feces. *Applied Environmental Microbiology* **81(19):**6749-6756.

**Supplementary Table S3.** Primers used for mRNA analysis.

| Target | Forward primer (5’ to 3’) | Reverse primer (5’ to 3’) |
| --- | --- | --- |
| IL-1β | TCGCTCAGGGTCACAAGAAA | CATCAGAGGCAAGGAGGAAAAC |
| TNF-α | AAATGGGCTCCCTCTCATCAGTTC | TCTGCTTGGTGGTTTGCTACGAC |
| NF-kB | GGA TGG TGA GGT CAC TCT | TCC TGA ACT CCA GCA CTC TCT TC |
| ATF4* | GAGCTTCCTGAACAGCGAAGTG | TGGCCACCTCCAGATAGTCATC |
| CHOP | CCTAGCTTGGCTGACAGAGG | CTGCTCCTTCTCCTTCATGC |
| PG | ATGAAGACCATTTACTTTG | CGGTTCCTCTTGGTGTTCATCAAC |
| ZO-1 | ACCCGAAACTGATGCTGTGGATAG | AAATGGCCGGGCAGAACTTGTGTA |
| Occ | ATGTCCGGCCGATGCTCTC | TTTGGCTGCTCTTGGGTCTGTAT |
| F4/80 | TGACAACCAGACGGCTTGTG | CAGGCGAGGAAAAGATAGTGT |
| MCP-1 | CAAGCAGAAGTGGGTTCAGGAT | TCTTCGGAGTTTGGGTTTGC |
| VCAM-1* | GTCACGGTCAAGTGTTTGGC | AGATCCGGGGGAGATGTCAA |
| RPL19 | GAAGGTCAAAGGGAATGTGTTCA | CCTTGTCTGCCTTCAGCTTGT |

IL-1 β; interleukin-1β, TNF-α; tumor necrosis factor alpha, NF-*k*B; nuclear factor kappa B, ATF4; activating transcription factor 4, CHOP; CCAAT/enhancer binding protein homologous protein, PG; proglucagon, ZO-1; zonula occludens-1, Occ; occludin, F4/80; macrophage F4/80 receptor, MCP-1; monocyte chemoattractant protein-1, VCAM-1, vascular cell adhesion molecule 1, RPL19; ribosomal protein L19.

**Supplementary Table S4.** Parameters of host physiology and serum biomarkers. Medians and interquartile ranges are provided. Those parameters or biomarkers that showed statistical significant difference are boldface for better recognition.

| Parameter/biomarker | Obese controls | Obese cherry-supplemented | Lean controls | P value |
| --- | --- | --- | --- | --- |
| **Body weight (g)** | **35.9^a^**  **(32.7-44.3) (n=10)** | **41.1^a,b^**  **(35.7-47.6) (n=12)** | **30.9^c^**  **(29.2-33.6) (n=10)** | **0.002** |
| **BMIs** | **4.4^a^**  **(4.1-4.8)**  **(n=10)** | **4.5^a,b^**  **(4.0-4.9)**  **(n=12)** | **3.3^c^**  **(3.1-3.5) (n=10)** | **<0.001** |
| **Weight cecum contents (mg)** | **191^a^**  **(104-234)** | **314^a,b^**  **(198-439)** | **128^a,c^**  **(93-152)** | **0.003** |
| Relative thickness of outer colon wall | 0.64 (0.6-0.7) | 0.73 (0.7-0.8) | 0.72 (0.6-0.7) | 0.08 |

Different letters state statistical significance difference.

**Supplementary Table S5.** mRNA levels of biomarkers involved in inflammation, cellular stress, and gut barrier function in colonic mucosal cells.

| Genes | mRNA levels/RPL19 mRNA | Obese controls | Lean controls | Obese cherry-supplemented |
| --- | --- | --- | --- | --- |
| Inflammation/ Cellular stress | IL-1β | 3.42  (1.0; 39.2) | 19.35  (5.6; 55.9 | 8.61  (2.5; 23.1) |
|  | TNF-α | 3.34  (1.2; 22.3) | 6.86 (3.1; 9.1) | 6.59  (1.1; 12.7) |
|  | NF-kB | 7.13  (1.6; 18.9) | 10.66  (6.4; 17.1) | 7.06  (4.1; 31.2) |
|  | ATF4* | 4.10  (1.1; 8.2) | 5.85  (2.7; 10.2) | 3.33  (1.7; 6.2) |
|  | CHOP | 6.58  (1.0; 9.9) | 7.73  (2.5; 11.2) | 6.79  (2.2; 9.2) |
| Intestinal permeability and gut barrier function | PG | 8.07  (1.8; 28.4) | 5.2  (1.1; 14.6) | 7.45  (2.6; 21.2) |
|  | ZO-1 | 3.92  (3.1; 5.6) | 3.69  (1.7; 6.8) | 3.70  (1.1; 5.5) |
|  | Occ | 4.38  (1.1; 6.5) | 3.24  (2.6; 5.3) | 3.40  (2.1; 3.8) |
| Monocyte infiltration/Cell adhesion/Inflammation | F4/80 | 4.61  (1.4; 11.3) | 9.49  (6.4; 28.6) | 5.04  (2.6; 26.5) |
|  | MCP-1 | 6.84  (1.2; 28.5) | 7.8  (4.0; 16.4) | 8.7  (1.2; 27.1) |
|  | VCAM-1 * | 6.42  (1.0; 32.0) | 8.51  (3.8; 12.0) | 3.98  (2.6; 7.0) |

ATF4: activating transcription factor 4, CHOP: CCAAT/enhancer binding protein homologous protein, IL-1β: interleukin 1β, Occ: occluding, PG: proglucagon, TNF- α: tumor necrosis factor- α; ZO-1, zonula occludens-1, F4/80: macrophage F4/80 receptor. Values of fold expression are median, range. *Outlier detection: http://www.miniwebtool.com/outlier-calculator. Data are median (min, max). Data was analyzed with Kruskal-Wallis test, *, p <0.05.

**Supplementary Table S6.** mRNA levels of biomarkers involved in inflammation, cellular stress, and gut barrier function in colon tissues.

| Genes | mRNA levels/RPL19 mRNA | Obese controls | Lean controls | Obese cherry-supplemented |
| --- | --- | --- | --- | --- |
| Inflammation/ Cellular stress | IL-1 | 65.81  (1.8; 295.3) | 86.89  (1.9; 154.6) | 141.90  (1.1; 309.7) |
|  | TNF-α | 150.10 (1.2; 384.2) | 253.70  (2.7; 326.9) | 318.90  (15.0; 382.1) |
|  | NF-κB | 46.86  (1.1; 192.0) | 57.27  (1.6; 88.6) | 93.31  (1.1; 142.2) |
|  | ATF4 | 9.82  (1.1; 33.3) | 12.57  (5.4; 17.5) | 17.12  (2.0; 29.5) |
|  | CHOP | 18.03  (2.2; 110.3) | 41.85  (1.2; 124.6) | 60.95  (1.6; 97.0) |
|  | IL-8 | 85.41  (1.6; 240.6) | 107.90  (3.9; 177.3) | 178.50  (70.3; 256.2) |
| Intestinal permeability and gut barrier function | OCC | 45.18  (1.4; 280.7) | 63.11  (1.6; 124.4) | 98.61  (5.3; 173.6) |
| Monocyte infiltration/Cell adhesion/Inflammation | F4/80 | 31.01  (1.1; 79.0) | 42.40  (2.9; 68.1) | 65.06  (1.2; 98.1) |
|  | VCAM1 | 12.70  (3.0; 46.5) | 21.86  (1.5; 29.9) | 31.38  (2.0; 69.7) |

IL: interleukin; TNF- α: tumor necrosis factor- α; NF-κB: nuclear factor kappa B transcription factor; ATF4: activating transcription factor 4; CHOP: CCAAT/enhancer binding protein homologous protein; OCC: occluding; VCAM1: vascular cell adhesion molecule 1. Data are median (min; max). Outlier detection: GraphPad Prism 6.
